# Gut Feelings Get Intense Due to Early Life Stress and Immune Imbalance

**DOI:** 10.1016/j.jcmgh.2025.101675

**Published:** 2025-11-04

**Authors:** Beatriz Thomasi

**Affiliations:** Department of Physiology, Michigan State University, East Lansing, Michigan

Irritable bowel syndrome (IBS) affects between 4% and 15% of humans and causes abdominal pain and altered bowel habits, with substantial impacts on quality of life and over $1 billion in annual United States health care costs.^1^ IBS is heterogeneous and classified by predominant bowel patterns such as constipation, diarrhea, mixed patterns, or unclassified. Although the pathophysiology of IBS remains unclear, it is notably influenced by stress,^2^ and key contributors to visceral hypersensitivity and dysmotility include immune imbalance and low-grade mucosal inflammation.^2^, ^3^, ^4^ Stress in the postnatal period is particularly important and early life stress is strongly correlated with IBS.^5^ Experiencing adverse psychosocial events in this critical period of neurodevelopment holds enormous plastic potential for the nervous system that, once modified, triggers altered intercellular communications that can manifest pathologically later in life.

In this issue of *Cellular and Molecular Gastroenterology and Hepatology*, Duan et al^6^ explore how early life adversity, intestinal microinflammation, and neuroimmune interactions culminate in IBS-like gut dysfunction in mice. Sympathetic gut signaling coordinates multiple metabolic pathways and immunomodulation, is imbalanced in patients with IBS, and such dysfunction is mimicked in rodents exposed to maternal separation.^7^^,^^8^ Extending their prior findings of eosinophil-mediated gastric microinflammation,^9^ the authors now report a similar eosinophilic profile in the large intestine mucosa, which is independent of classical inflammatory markers. Elevated serum norepinephrine and the suppression of eosinophil infiltration by sympathetic blockades highlight a key role for sympathetic overactivity in gut immune imbalance.

To critically address the potential mechanisms of sympathetic-eosinophil interactions, the authors used genetic tools to selectively activate peripheral sympathetic neurons. Using specific adeno-associated virus with site-specific injections, hM3Dq expression was induced in the celiac superior mesenteric ganglion and preganglionic spinal neurons. Adeno-associated viral expression and stimulation of hM3Dq receptors in mice reproduced the elevated serum norepinephrine and colonic eosinophilia observed in adult rats following maternal separation. Furthermore, transfected mice containing hM3Dq demonstrated visceral hypersensitivity following chemogenetic receptor activation, directly connecting sympathetic nervous system (SNS) stimulation and norepinephrine release with eosinophil recruitment and nociception. Additional support for this concept came from the observation that colorectal norepinephrine infusion upregulates *Ccl11* gene expression, which encodes eotaxin-1, a chemoattractant released by multiple cell types that regulates intestinal inflammation.^10^ Here, eosinophil recruitment occurs via upregulation of eotaxin-1 by mesenchymal cells (likely fibroblasts) through both α- and β-adrenergic signaling. A key contribution of this study is the demonstration that eotaxin-1 is essential for SNS-induced eosinophil infiltration and colon hypersensitivity in adult mice exposed to maternal separation. Although this mechanism is not fully characterized in IBS, fibroblast-driven immune activation and tissue remodeling are well-described in inflammatory bowel diseases and may represent a promising therapeutic target.^11^ Notably, blockade of eosinophil recruitment and eotaxin-1 release suppressed visceral hypersensitivity, highlighting the translational potential of this approach.

Duan and colleagues delineated a novel pathway in which eosinophils are recruited following SNS overactivation to drive visceral hypersensitivity using an elegant combination of pharmacological treatments targeting adrenergic receptors, cell labeling, flow cytometry, and adenoviral vectors. Some limitations of this work arise from challenges integrating these findings into our understanding of the complex interactions of the multiple intestinal layers, such as the mucosa, submucosa, and neural plexuses. Norepinephrine may drive neuroplastic changes inside the myenteric and submucosal plexus where the extrinsic innervation reaches the gut and nociceptive transduction occurs. Muscularis macrophages and enteric glia are also integral parts of this puzzle.^12^^,^^13^ However, because chemotactic chemokines are elevated in patients with IBS and play a fundamental role in recruiting immune cells and microinflammation,^14^ description of eotaxin-1 as a key mechanism represents a step forward in understanding the neuroimmune mechanisms of IBS. Furthermore, the data using human colorectal biopsies from patients with IBS exhibited increased numbers of myelin basic protein+ cells, along with eotaxin-1+-vimentin+ double-labeled cells, further supporting the translational relevance of this pathway. By outlining a multistep mechanism linking stress, neuroimmune signaling, and IBS-like symptoms, the work offers new avenues to test therapeutic strategies for alleviating stress-related gut dysfunction.
